# The prevalence of functional dyspepsia using Rome IV questionnaire among chronic kidney disease patients

**DOI:** 10.1080/0886022X.2024.2344651

**Published:** 2024-04-24

**Authors:** Omar Abdelazim, Alshymaa A. Hassnine, Basma Fathy, Ahmed Mgdy, Nady Semeda, Shereen R. Mahmoud, Zeinab M. Saad, Haitham A. Mahmoud

**Affiliations:** a Department of Tropical Medicine, Minia University, Egypt; b Department of Internal Medicine, Minia University, Egypt

**Keywords:** CKD, functional dyspepsia, EPS, ROME IV questionnaire

## Abstract

**Background**: Symptoms of dyspepsia are usually encountered by chronic kidney disease patients. Abdominal discomfort is commonly seen in CKD patients with no other causes of organic affection. **Aim:** to determine the prevalence of functional dyspepsia in CKD patients, and which subtype is predominant in them. **Materials and patients:** This observational study included 150 CKD patients. Clinical and laboratory data were recorded for every patient. All the patients were interviewed using the ROME IV questionnaire of functional dyspepsia. Patients fulfilling criteria for functional dyspepsia were exposed to upper GI endoscopy. **Results:** Overall, 73 (48.7%) of CKD patients were males and 77 (51.3%) were females with mean age of (45.71 ± 9.59) and mean BMI (26.58 ± 5.39). The frequency of functional dyspepsia among CKD patients was determined to be 14.7% (22 out of 150 patients). Among those affected by functional dyspepsia, the most prevalent subtype was found to be Epigastric Pain Syndrome (EPS), accounting for 59% (13 out of 22 cases). The most common predictor of FD in CKD patients was chronic HCV infection, hemodialysis, stage of CKD and eGFR as revealed by Univariate regression analysis. **Conclusion:** The prevalence of FD amongst CKD patients is 14.7% with EPS the predominant subtype. Male patients, HCV patients, patients with higher CKD stages and highly impaired eGFR (low eGFR) are more probable to have FD.

## Introduction

Chronic kidney disease is well-defined as organic or functional changes in renal function for a period of time (at least 3 months) with implications on the health [1], and it is prevalence is high all over the world [1].

The National Kidney Foundation [Kidney Dialysis Outcomes Quality Initiative] categorized CKD into 5stages contingent on the estimated GFR [2].

The stages of CKD are classified as follows^:^

- Stage 1: Kidney damage with normal or increased GFR (>90 mL/min/1.73 m [2])
- Stage 2: Mild reduction in GFR (60-89 mL/min/1.73 m [2])
- Stage 3a: Moderate reduction in GFR (45–59 mL/min/1.73 m [2])
- Stage 3b: Moderate reduction in GFR (30–44 mL/min/1.73 m [2])
- Stage 4: Severe reduction in GFR (15–29 mL/min/1.73 m [2])
- Stage 5: Kidney failure (GFR < 15 mL/min/1.73 m [2] or dialysis)

Dyspepsia which means poor digestion indicates an upper GIT syndrome triggered *via* food ingestion. Dyspepsia includes abdominal fullness after eating as well as early satiety in addition to also epigastric burning or pain which may not related to food ingestion.

It may present as burning pain, nausea, bloating, and fullness after meals, a feeling of indigestion or slow digestion. It may be ulcer disease or functional dyspepsia i.e. non-ulcer dyspepsia [3].

Due to uremia, manifestation of gastritis, peptic ulcer disease or mucosal ulcerations at any part of gastrointestinal tract leading to upper abdominal pain, nausea, vomiting or GI bleeding is common. Elevated gastrin levels have also been found in such patients. This, along with uremia, inflammation and local circulatory disturbances may lead to injury to mucosa. CKD patients have greater prevalence of gastric mucosal injury than normal ­population [4].

Functional dyspepsia is defined as the presence of symptoms which are thought to originate from gastro duodenal region, in the lack of any organic, systemic or metabolic disease that is likely to clarify the symptoms **[**5]. It is also referred to as “Dyspepsia Symptom Complex” and has been divided into; Post prandial Distress Syndrome (PDS) and Epigastric Pain Syndrome (EPS).

Numerous resarches done in Europe, North America and Oceania have revealed that the prevalence rate of dyspepsia in broad-spectrum inhabitants is between 3% to 40% and these variants of the prevalence rates are owing the alteration definition used. Strid et al. studied the relation among dyspeptic symptoms in patients in dialysis and pre-dialysis programs. They reported increased dyspeptic symptoms between patients in hemodialysis [6].

The prevalence of dyspepsia amongst hemodialysis patients varies among 48% and 70% [7]. A current well done meta-analysis in addition to Cochrane Database systematic reviews display that there is a small however significant benefit of reducing H pylori in patients with dyspepsia **[**8].

## Patients & methods

This cross-sectional study was conducted at Hospital of Minia University, over a period of nine months from January 2020 to September 2020. 150 patients with chronic kidney disease were enrolled in our research.

### Inclusion criteria

1. Patients of CKD irrespective of its stage.
2. Age > 18 years.
3. Gender: both males and females are involved.
4. Patients willing to participate in study.

### Exclusion criteria

1. Presence of disorders or clinical situation that could avoid patients from answering the questionnaires.
2. Patients not agreeable for upper GI endoscopy.
3. Patients with recognized gastric disease.
4. Patients with alarm feature (red flags):
5. Loss of Weight.
6. Chronic Vomiting.
7. Odynophagia.
8. Dysphagia.
9. History of gastrointestinal bleeding.
10. History of upper GIT malignancy.
11. Patients with other chronic systemic disorders [e.g., heart failure, Gall blader disease, etc]

After getting agreement from university’s ethics committee, subjects fulfilling inclusion criteria were enrolled into the research. A written knowledgeable consent was obtained from all participants.

All the CKD patients admitted in medicine wards/dialysis unit were included in the study. CKD was diagnosed on basis of history, blood urea and serum creatinine level, US findings kidney size, cortico medullary differentiation and cortical thickness. Demographic data was collected in all patients. Patients were screened for anemia diabetes and hypertension. Laboratory investigations in form of complete blood count and kidney function tests were done in every patient. eGFR was calculated as per CKD-EPI formula, taking the values of serum creatinine into consideration and patients were categorized according to 5 stages of CKD.

All eligible participants who provided consent for inclusion in the study were interviewed using the ROME IV questionnaire, which was appropriately translated into the vernacular language (Arabic) to accommodate non-English speaking subjects. The interviews were conducted in the participants’ native language to ensure correct understanding and effective communication. The validated Rome IV diagnostic questionnaire for FD was performed *via* the Rome committee.

The FD module was taken from the Rome Foundation website (http://www.romecriteria.org/questionnaires/).

Functional dyspepsia includes the following subtypes:

a. Postprandial Distress Syndrome

Must fulfill at least one of the following at least 3 days a week for the last 3 months:

1. Bothersome postprandial fullness (i.e., severe sufficient to effect on habitual activities)
2. Bothersome early satiation (i.e., severe to prevent finishing regular size meal)
3. No evidence of organic, systemic, or metabolic disorders that is likely to clarify the symptoms on routine investigations (including at upper endoscopy)

b. Epigastric Pain Syndrome

Must fulfill the subsequent criteria at least 1 day a week for the last 3 months:

1. Bothersome epigastric pain (i.e., severe sufficient to impact on usual activities) OR
2. Bothersome epigastric burning (i.e., severe enough to impact on usual activities)
3. No indication of organic, systemic, or metabolic disease that is likely to explain the symptoms on routine investigations (including at upper endoscopy).
4. Symptoms onset at least 6 months preceding to diagnosis

### Statistical analysis

All analysis were done using SPSS version 20. Quantitative data were obtainable *via* mean, standard deviation while qualitative records were presented *via* frequency distribution. The Chi-square test was used to compare among proportions or Fisher exact test “if >20 of cells had predictable count less than 5”. Independent sample *t-*test was used to compare two means.

Logistic regression analysis was used to predict the outcome of dissimilar independent variables on the target (dependent variable). The probability of less than 0.05 was used as a cut off point for significant tests.

## Results

There was a significant difference between CKD patients with FD and CKD patients without FD as regard to sex (p value < 0.047) and HCV infection (p value 0.001) but there was no significant difference as regard to Age, Residence, educational level, Smoking, BMI and history of DM or HTN as shown in Table 1.

**Table 1.** Socio-demographic data among the studied cases.

Variable	Patients with FD (*N* = 22) N (%)	Patients without FD (*N* = 128) N (%)	*p* value
Age (years)			
Mean ± SD	48.31 ± 10.52	45.27 ± 9.39	0.44
Sex			
Male	15 (68.2%)	58 (45.3%)	0.047*
Female	7 (31.8%)	70 (54.7%)	
Residence			
Rural	15 (68.2%)	88 (68.8%)	0.96
Urban	7 (31.8%)	40 (31.2%)	
Educational level			
not educated	4(18.1%)	20(15.6%)	0.22
Low	8(36.3%)	41(32%)	
High	10(45.4%)	67(52.3%)	
BMI			
Mean ± SD	27.19 ± 4.79	26.47 ± 5.51	0.36
Smoking status			
Non smoker	16 (72.7%)	90 (70.3%)	0.82
Smoker	6 (27.3%)	38 (29.7%)	
DM			
Negative	14 (63.6%)	65 (50.8%)	0.27
Positive	8 (36.4%)	53 (49.2%)	
HTN			
Negative	11 (50%)	54 (42.2%)	0.49
Positive	11 (50%)	74 (57.8%)	
HCV			
Negative	9 (40.9%)	98 (76.6%)	0.001*
Positive	13 (59.1%)	30 (23.4%)	

There was no significant difference between CKD patients with FD and CKD patients without FD as regard to laboratory parameters or sonographic findings as found in Table 2.

**Table 2.** Laboratory investigations and sonographic findings among the studied cases.

Variable	Patients with FD (*N* = 22) Mean ± SD	Patients without FD (*N* = 128) Mean ± SD	*p* value
Hb	10.10 ± 2.75	10.29 ± 4.94	0.97
TLC	7.75 ± 3.81	6.95 ± 3.32	0.21
PLT	178.68 ± 81.04	213.17 ± 88.34	0.13
Urea	132.31 ± 40.32	135.14 ± 25.21	0.26
Creatinine	5.37 ± 2.05	5.56 ± 0.95	0.41
Kideny size			
Small	8 (36.4%)	67 (52.3%)	0.31
Malrotated	1 (4.5%)	7 (5.5%)	
Average	13 (59.1%)	54 (42.2%)	
Echogenicity			
Normal	1 (4.5%)	2 (1.6%)	
Grade 1–2	8 (36.4%)	39 (30.5%)	0.55
Grade 3–4	6 (27.3%)	44 (34.4%)	
Altered	7 (31.8%)	43 (33.6%)	
Other findings			
No backpressure	10 (45.5%)	53 (41.4%)	0.98
Minimal backpressure	7 (31.8%)	36 (28.1%)	
Gravels	2 (9.1%)	11 (8.6%)	
Stones	2 (9.1%)	18 (14.1%)	
Cyst	1 (4.5%)	10 (7.8%)	

There was a significant difference between CKD patients with FD and without FD as regard to stage of CKD with (p value <0.001). It was found that stage 5 CKD had a higher prevalence among individuals with FD, accounting for 77.3% (17 out of 22) of the cases. However, no significant difference was found in terms of the mean and standard deviation of (eGFR) between the two groups. It also shows that there was a significant difference between 2 groups as regard to Dialysis and vascular access with (p value < 0.001) for both variables as presented in Table 3.

**Table 3.** Relation of CKD stages, e GFR, dialysis and vascular access with functional dyspepsia for characteristics of illness among the studied cases.

Variable	Patients with FD (*N* = 22) N (%)	Patients without FD (*N* = 128) N (%)	*p* value
CKD			
Grade 3b	4 (18.2%)	2 (1.6%)	0.001*
Grade 4	1 (4.5%)	3 (2.3%)	
Grade 5	17 (77.3%)	123 (96.1%)	
Estimated GFR			
Mean ± SD	14.67 ± 10.72	10.79 ± 4.34	0.18
Dialysis			
No	8 (36.4%)	12 (9.4%)	0.001*
Yes	14 (63.6%)	116 (90.6%)	
Vascular access			
No	8 (36.4%)	12 (9.4%)	0.001*
AV shunt	14 (63.6%)	116 (90.6%)	

The prevalence of functional dyspepsia among CKD patients was determined to be 14.7% (22 out of 150 patients) as illustrated in Figure 2.

**Figure 1.**
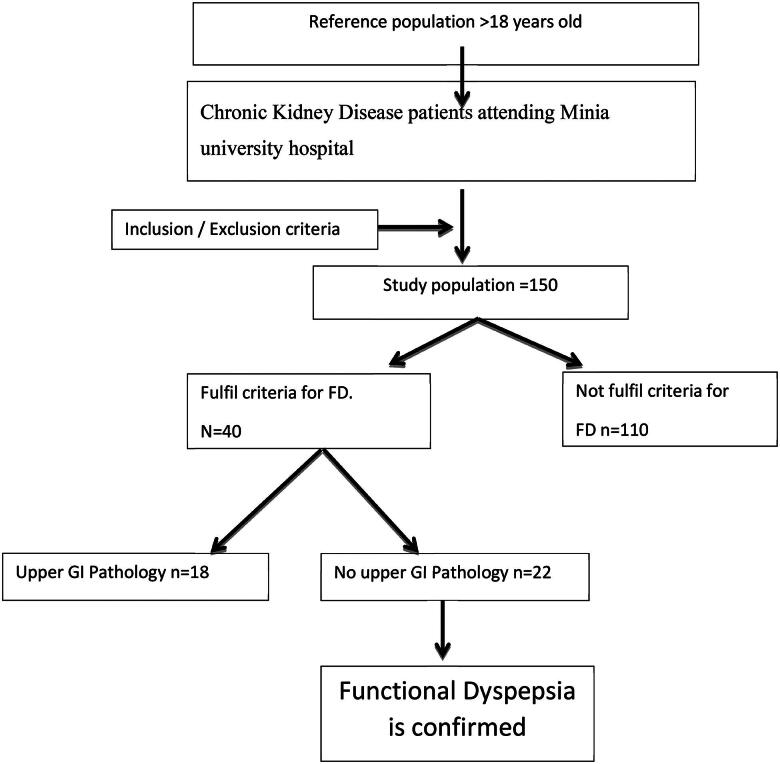
Flow chart study population.

**Figure 2.**
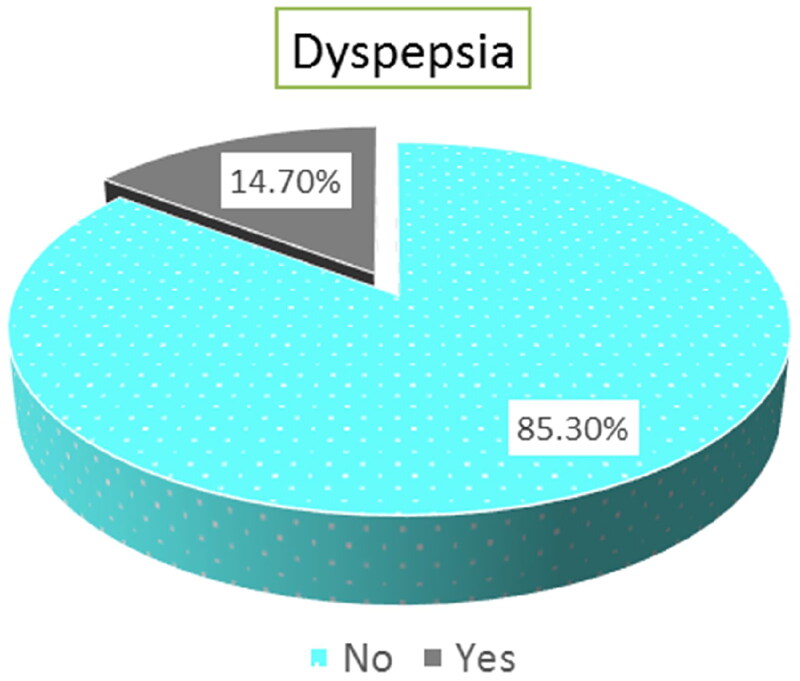
Frequency of Functional dyspepsia among the studied patients (*N* = 150).

**Figure 3.**
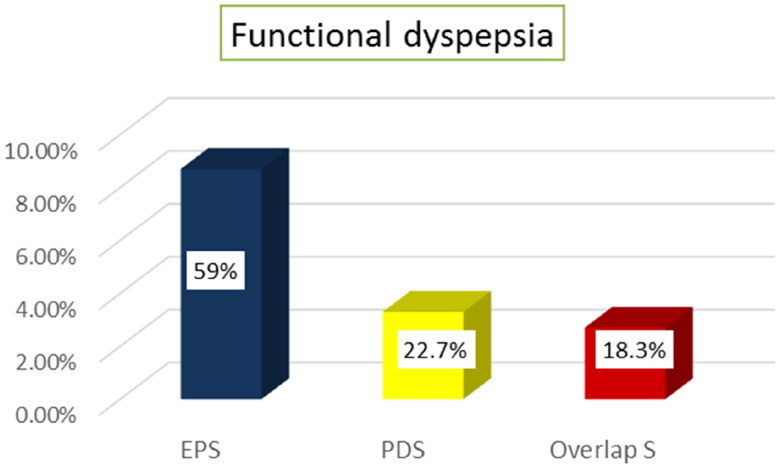
Prevalence of functional dyspepsia subtypes (*N* = 22).

Regarding functional dyspepsia subtypes, EPS was the predominant subtype with 13/22 patients(59%) as illustrated in Figure 3.

Univariate regression analysis of factors associated with functional dyspepsia patients was done. The most important predictors of FD in CKD patients were HCV infection, hemodialysis, stage of CKD and eGFR with p value (>0.001) (>0.001) (>0.01) (>0.01) respectively.

There was no significant association between age, sex, residence, smoking, educational level, BMI, DM, HTN or drug history and FD as found in Table 4.

**Table 4.** Univariate analysis for factors associated with functional dyspepsia among CKD patients.

Independent factors	Crude OR (95% CI)	*p* value
Age (years)	1.03 (0.99–1.1)	0.17
Sex		
Male	1 (Ref)	0.05
Female	2.59 (9.9–6.7)	
Residence		
Rural	1 (Ref)	0.96
Urban	0.97 (0.37–2.57)	
Educational level		
Not educated	1 (Ref)	0.98
Low	1.01 (0.38–2.67)	
High	1.03(0.65–2.51)	
BMI	1.02 (0.94 − 1.12)	0.57
Smoking status		
Non smoker	1 (Ref)	0.82
Smoker	1.13 (0.41–3.09)	
DM		
-ve	1 (Ref)	
+ve	1.69 (0.66–4.3)	0.27
HTN		
-ve	1 (Ref)	0.49
+ve	1.37 (0.55 − 3.39)	
HCV		
-ve	1 (Ref)	0.001*
+ve	0.21 (0.08–0.54)	
Stages CKD		
G3b	1 (Ref)	0.01*
G4	14.47 (2.46 − 85.08)	
G5	2.41 (0.24–24.52)	
E GFR	1.08 (1.02–1.15)	0.01*

## Discussion

Dyspeptic symptoms are frequently experienced by patients diagnosed with chronic kidney disease (CKD), particularly among those undergoing hemodialysis [5]. Patients with CKD often exhibit a variety of gastrointestinal symptoms, with abdominal pain or discomfort being commonly observed in clinical practice, even in the absence of other systemic complications. Many patients attribute these abdominal complaints to functional reasons [7].

In comparison to the general population, patients with CKD are more prone to developing gastric mucosal injuries which may be attributed to factors such as local or systemic circulatory insufficiency, hypergastrinemia, hyperammonemia, and inflammation [4].

The relationship between functional dyspepsia and CKD has not been extensively investigated. This knowledge gap has prompted our interest in exploring the potential association between functional dyspepsia and chronic kidney disease.

In our research age is not a predictive issue for functional dyspepsia. This matches with the findings from previous studies reported by [9,10 ]who institute that age was not predictive of FD **[**9]. However, **[**11]found that the prevalence of FD was peak at the age of 41 to 50 years old **[**11]. No relation was found with educational level or residence in our study. Comparable results regarding education level were established *via* Aro et., al in their study **[**10].

Little research has investigated the association between gender and functional dyspepsia (FD) in the general population. In our study, we observed a higher prevalence of FD in males CKD patients, compared to female patients. However, this finding contradicts the results reported by Young Sun Kim and Na young Kim in their 2020 study. They found that FD is more commonly observed in women compared to men. In addition, they explored various aspects, including prevalence, clinical manifestations, and quality of life in FD, emphasizing the importance of understanding the distribution and burden of the disorder, evaluating treatment options, and developing new therapeutic strategies [12]. Further research is required to clarify the role of gender in FD and its implications for clinical practice.

In a recent meta-analysis performed by Ford et al. in 2016, the prevalence of functional dyspepsia (FD) was examined in 55 studies, specifically considering gender differences. The analysis revealed a slightly higher pooled prevalence of FD in women compared to men, with rates of 25.3% and 21.9%, respectively [13]. These findings contrast with our study results, which indicated a higher prevalence of FD in males among CKD patients. It is important to consider that our study focused on a selective cluster of CKD patients, which may result in variations in age and gender compared to the general population. Additionally, the limited number of cases and the relatively short duration of the study may introduce a false impression regarding the factors influencing FD. Therefore, it is crucial to conduct further studies with a broader scope and an increased number of cases to gain a more comprehensive understanding of the association between FD, gender, and other potential influencing factors.

A study by Salles et, al. (2013) found that functional dyspepsia had no relation to BMI in chronic kidney disease patients. Similar findings were described in our research **[**14].

Our study found no significant association between Functional Dyspepsia (FD) and the presence of diabetes mellitus (DM) or hypertension (HTN) in patients with chronic kidney disease (CKD). These results are consistent with previous research conducted by Bacci and Chehter in 2013 [1] and Salles et al. in 2013 [14]. Both studies also reported no significant relationship between FD and DM or HTN in CKD patients.

Furthermore, our study revealed no correlation between FD and hemoglobin levels, total leukocyte count, or platelet count. These results agree with the findings of Salles et al. in 2013, who similarly found no association between dyspepsia and hemoglobin levels [14].

In summary, our research supports the notion that there is no significant link between FD and the presence of DM or HTN in CKD patients, as well as no association between FD and hemoglobin levels, total leukocyte count, or platelet count. These findings contribute to the existing body of literature on the topic and provide further insights into the factors influencing FD in CKD patients.

In our research, we observed a significant association between the deterioration of renal function and the presence of functional dyspepsia. Specifically, among patients with Stage 5 chronic kidney disease (CKD), a higher prevalence of functional dyspepsia was noted, with 17 out of 22 patients (77.3%) exhibiting this condition. This finding contradicts the results identified by Manohar and Baxy, who did not report a correlation between the two variables in their previous study [15]. Furthermore, Bacci and Chehter reported that worsening creatinine levels did not emerge as a risk factor for the development of dyspepsia or functional dyspepsia subtypes [1]. It is worth noting that the target sample size in the study performed by Manohar and Baxy was approximately 40, which may have influenced the prevalence of functional dyspepsia and its relationship with the deterioration of kidney function.

A significant relation was reported among e GFR in addition to functional dyspepsia in our research. This finding is compatible with the reseach showed *via* Bacci and Chehter who reported that patients with a high e GFR are at higher chances of developing functional dyspepsia **[**1].

Functional Dyspepsia is uncommon in hemodialysis **[**16]. Between 130/150 hemodialysis patients only 14 (10.7%) were diagnosed functional dyspepsia. This could be clarified *via* the fact that chronic renal failure itself is a risk factor for organic dyspepsia by reason of associated metabolic disturbance in CKD and unhealthy gastric mucosa. This is compatible with a preceding research done *via* [16] which discovered that prevalence of FD was 1.5% amongst hemodialysis patients **[**16]. However, our findings disagree with findings of preceding researches done *via* Bacci and Chehter plus additional research *via* Manohar and Baxy which discovered that FD is more prevalent in hemodialysis patients (30%,22.5%) respectively **[**1, 15]. This could be described through the fact that they had worked on a very heterogeneous sample involved of hemodialysis patients, chronic renal failure non-hemodialysis plus non-renal failure patients besides the small sample size (40 patients) in Manohar and Baxy study.

Through using univariate analysis, we used multiple factors (Age, sex residence, educational level, BMI, smoking, history of DM HTN or HCV infection, stages of CKD and e GFR) to notice the independent factors that affect happening of FD. We found that the association of HCV infection, stage of disease and low e GFR to be independent factors related with FD. Bacci and Chehter study reported age to be independent factor in their study **[**1].

Definitions and Diagnostic criteria affect the prevalence rates of FD. To our best Knowledge, this is the first research evaluating FD as well as its subtypes amongst CKD patients using ROME IV criteria (the latest review of ROME foundation).

There were some limitations of our research, Firstly some of the patients in the research had been cured for their dyspeptic symptoms and this would alteration the course of symptom and could influence the true prevalence of FD. Also, it was impossible to make longitudinal follow up of patients to detect the probable prompting factors for FD in a timely method because of the high mortality rates related to hemodialysis.

## Conclusion

The prevalence of FD amongst CKD patients is 14.7% with EPS the predominant subtype. Male patients, HCV patients, patients with higher CKD stages and highly impaired eGFR (low eGFR) are more likely to have FD.

## Data Availability

The article contains all the data produced or analyzed during this research
